# *De novo* design of programmable inducible promoters

**DOI:** 10.1093/nar/gkz772

**Published:** 2019-09-25

**Authors:** Xiangyang Liu, Sanjan T P Gupta, Devesh Bhimsaria, Jennifer L Reed, José A Rodríguez-Martínez, Aseem Z Ansari, Srivatsan Raman

**Affiliations:** 1 Department of Biochemistry, University of Wisconsin-Madison, Madison, WI 53706, USA; 2 The Great Lakes Bioenergy Research Center, University of Wisconsin-Madison, Madison, WI 53706, USA; 3 Department of Chemical and Biological Engineering, University of Wisconsin-Madison, Madison, WI 53706, USA; 4 Department of Biology, University of Puerto Rico-Rio Piedras, San Juan, PR 00925, USA; 5 The Genome Center of Wisconsin, University of Wisconsin-Madison, Madison, WI 53706, USA; 6 Department of Bacteriology, University of Wisconsin-Madison, Madison, WI 53706, USA

## Abstract

Ligand-responsive allosteric transcription factors (aTF) play a vital role in genetic circuits and high-throughput screening because they transduce biochemical signals into gene expression changes. Programmable control of gene expression from aTF-regulated promoter is important because different downstream effector genes function optimally at different expression levels. However, tuning gene expression of native promoters is difficult due to complex layers of homeostatic regulation encoded within them. We engineered synthetic promoters *de novo* by embedding operator sites with varying affinities and radically reshaped binding preferences within a minimal, constitutive *Escherichia coli* promoter. Multiplexed cell-based screening of promoters for three TetR-like aTFs generated with this approach gave rich diversity of gene expression levels, dynamic ranges and ligand sensitivities and were 50- to 100-fold more active over their respective native promoters. Machine learning on our dataset revealed that relative position of the core motif and bases flanking the core motif play an important role in modulating induction response. Our generalized approach yields customizable and programmable aTF-regulated promoters for engineering cellular pathways and enables the discovery of new small molecule biosensors.

## INTRODUCTION

The ability to program gene expression is a fundamental requirement for engineering new cellular functions. Development of standardized parts has enabled the design of sophisticated, model-guided genetic circuits with minimal human intervention (1). This heralds a new era of synthetic biology where living cells can be programmed to execute user-defined functions with high fidelity, and are more than just a ‘bag of enzymes’. Ligand-responsive allosteric transcription factors (aTF) are a vital component of genetic circuits because they control the flow of information by transducing biochemical signals into gene expression (2). aTFs convert internal or external cues (input) into ligand-inducible promoter expression (output) of downstream effector genes, providing versatile control of virtually any cellular process. Much effort has been directed toward expanding the suite of ligand inputs to build new aTF biosensors (3,4). However, our ability to program gene expression output from a promoter regulated by an aTF (native promoter) is poorly developed and has received far less attention. Programmable control of gene expression from an aTF-regulated native promoter is important because different downstream effector genes function optimally at different expression levels (5). For instance, high expression of a fluorescent reporter facilitates visualization and resolvability of cells for imaging (microscope or flow cytometer). Similarly, a weak metabolic enzyme may have to be overexpressed to improve pathway flux. In contrast, expression of a transporter or a recombinase has to be carefully controlled within a tight window as overexpression may be highly deleterious. In complex circuits such as timers, counters and memory devices with multiple aTF-based logic gates, gene expression output of each gate has to be correctly ‘matched’ to activate the next gate so as to effectively relay the signal (1,6,7). aTF biosensors used in metabolic pathway optimization can often be ineffective if their dynamic range is low or incompatible with intracellular metabolite concentrations. These examples illustrate the need for programmable aTF-regulated promoters that can be tailored for different downstream effector genes and applications.

**Figure 1.**
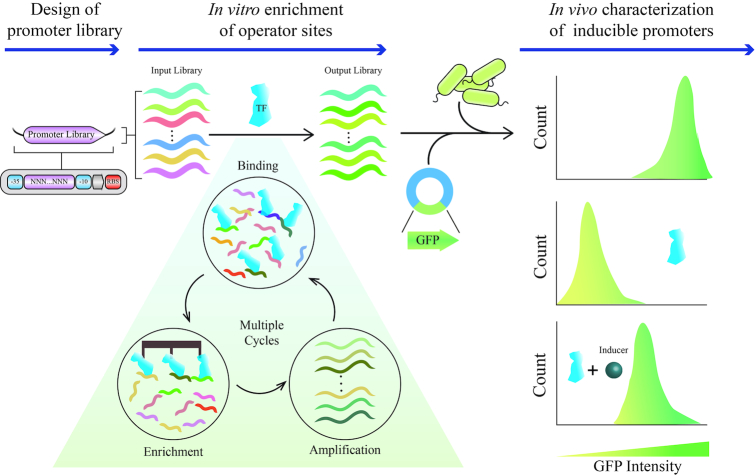
*De novo* promoter engineering scheme. Design workflow involves three steps: creating a promoter library with randomized bases between -35 and -10 sites of a constitutive *E. coli* promoter, enrichment of promoters that can bind to aTF by *in vitro* selection, and multiplexed screening for inducible promoters by high-throughput cell sorting followed by clonal testing.

Currently, we are limited to mostly one or a few well-characterized native promoters depending on the aTF. Since native promoters are evolutionarily optimized for the endogenous role of the aTF, they are saddled with sites for feedback control, co-regulators and may also contain elements that enhance context-dependence including sites for alternative sigma and stress-response factors. Because these features cannot be easily disentangled, modifying gene expression properties like baseline expression, dynamic range and dose-response is notoriously difficult in native promoters. Massively-parallel reporter assays have been developed to quantitatively characterize promoter-transcription factor interactions (8) and to engineer new promoters (9). These approaches mutagenize the native promoter and measure resultant changes in reporter expression to infer underlying sequence-function landscape and binding energetics (10). Instead of tinkering with native promoters, we decided to *de novo* engineer aTF-regulated promoters based on a minimal constitutive promoter completely unrelated to the native promoter and devoid of regulation. Our goal was to engineer a suite of inducible promoters for an aTF displaying the full range of gene expression levels and ligand response characteristics. We chose to engineer promoters for TetR-like family of aTFs because they are commonly used in genetic circuits (11) and represent a large pool of potential new biosensors for primary and secondary metabolites (12). TetR-like aTFs have a simple mechanism of transcription: when bound to their operator (binding site on DNA), they repress gene expression by sterically blocking RNA polymerase. Upon binding to a ligand, an allosteric conformational change dislodges the aTF from the operator allowing RNA polymerase to transcribe the downstream gene. This led us to hypothesize that gene expression could be modulated by changing the affinity of an aTF for its operator sequence as this would alter promoter occupancy by RNA polymerase. In other words, since equilibrium distributions of DNA-bound (unliganded) and DNA-unbound (liganded) aTFs would be different for operators with different aTF affinities, it would result in distinct ligand-induced transcriptional responses. We implemented this concept as follows (Figure 1). We generated a library of ∼10^5^ aTF operator sites of varying affinities by *in vitro* selection starting from randomized N-mers. The *in vitro* selected operators had radically reshaped binding architecture but with similar core motifs found in their respective native counterparts. The operator library was positioned as a spacer between -35 and -10 sites of a minimal constitutive *Escherichia coli* promoter to create a library of aTF-regulatable promoters. We then enriched for functional promoter variants with different gene expression levels by sorting cells carrying the promoter library driving GFP (without and with inducer) followed by clonal screening (Figure 1). For each aTF, this yielded greater than 30–50 inducible promoters with low baseline expression exhibiting a full range of ligand-induced gene expression spanning 5–90% of expression from the constitutive promoter. The engineered promoters also had a broad distribution of dynamic ranges, ligand sensitivities and cooperativity of response. We demonstrate the generalizability of this approach by engineering inducible promoters for three TetR-family aTFs—TtgR, PmeR and NalC. Statistical machine learning on our dataset revealed that although a core sequence motif was required for aTF binding, relative position of the core motif and bases flanking the core motif played an important role in modulating induction response. Our results show that a simple model of competitive access to the promoter between aTF and RNA polymerase can be exploited to generate a rich variety of ligand-induced gene expression levels and dose response functions. This approach could be useful for rapidly engineering promoters for new metagenomic aTFs sensing useful molecules, by sidestepping the need to identify and optimize their native promoters.

## MATERIALS AND METHODS

### In vitro enrichment of operator sites

We synthesized codon optimized genes for all three aTFs (Genscript, Inc) and cloned them using Gibson Assembly into overexpression vector pET-31b with a C-terminus His tag (15). We transformed sequence-verified plasmids into BL21(DE3) *E. coli* and plated them on Luria-Bertani (LB) plates containing 100 μg/ml carbenicillin. After overnight growth at 37°C, we inoculated colonies into 3 ml LB medium (100 μg/ml carbenicillin) and grew the cells to an optical density of 0.6 at 600 nm (OD_600_) at 37°C. Then we induced cultures with 0.5 mM isopropyl β-d-1-thiogalactopyranoside (IPTG) for 3 h and confirmed protein expressions on a 4–12% Bis–Tris glycine gel. Cells were subsequently lysed by sonication in a lysis buffer (50 mM HEPES pH 7.5, 300 mM NaCl, 5 mM BME 10% glycerol).

We combined equimolar amounts of 16, 17, 18 and 19 base pairs (bp) hybridized promoter libraries into one mixed library. The mixed library (100 nM) was incubated with 2 μl protein lysate in 1× enrichment buffer (15 mM Tris–HCl pH 7.5, 100 mM NaCl, 1 mM BME, 0.02 U poly-dI-dC, 0.03% BSA, 0.05% NP40) in 20 μl reactions at room temperature for 1 h. We mixed 2.5 μl magnetic His-tag pull-down beads (pre-washed three times with 500 μl buffer of 15 mM Tris–HCl pH 7.5, 100 mM NaCl, 0.03% BSA) with protein and DNA by rotating at low speed for 30 min at 4°C. We pulled down protein–DNA complex attached to beads, removed unbound DNA and washed the complex three times with pre-wash buffer (15 mM Tris–HCl pH 7.5, 100 mM NaCl, 0.03% BSA). After removing final wash buffer, we mixed the complex with Kapa HF PCR master mix and primers to amplify enriched DNA (PCR protocol: 95°C for 3 min, 98°C for 20 s, 60°C for 20 s, 72°C for 10 s. Repeat step 2 to 4 for 20 times, 72°C for 20 s) and confirmed the amplicon size on a 2% agarose gel.

To prepare *in vitro* enriched DNA for deep sequencing, we added standard Illumina P5 adaptor and custom 8 bp barcode to the 5′ end using PCR (PCR protocol: 95°C for 3 min, 98°C for 20 s, 60°C for 20 s, 72°C for 10 s. Repeat step 2 to 4 for 15 times, 72°C for 20 s). Barcoded DNA amplicon size was checked using 2100 Bioanalyzer (Agilent) and sequenced using Illumina HiSeq 2500 sequencing system (University of Wisconsin-Madison, Biotechnology Center DNA sequencing facility) after addition of 40% PhiX sequencing control (Illumina). The sequencing run gave around 289 million raw reads.

### Analysis of operator motifs using a Markov model

First reads from sequencing were demultiplexed using a 8 bp barcode and truncated to include only the N-mer random portion of the library. For the analysis, we use ‘gapped *k*-mers’ - these are *k*-mers with spaces in the middle of the form ‘*l*-mer *m*-space *n*-mer’ (thus *l* + *m* + *n* = *k*), where *l* and *n* can take values 4 or 5 and spacer m can take values 0 to *N* – *l* – *n*, where *N* is the length of the random portion of the library. For example, ACTACxxxACGC is a ‘gapped 12-mer’ of ‘5-mer 3-spacer 4-mer’ type, where spacers x can be any nucleotide A, C, G or T. First occurrence of such gapped k-mers is counted in the random portion of the sequenced sample and those with occurrence <50 counts are removed from further analysis. Then the enrichment for each gapped *k*-mer is calculated by dividing these counts in the sequenced sample against the expected number of occurrences of the same gapped *k*-mer from the starting random library (Bhimsaria *et al.* in preparation). For the starting library the sequencing depth is enough to have counts for all 8-mer (without spaces or gaps in the middle), but the depth (total number of read counts) isn’t enough to capture all gapped *k*-mers or even non gapped *k*-mers with *k* ≥10. Thus, to normalize the counts of gapped *k*-mer in the sample against library, a fifth-order Markov model was created to get the expected number of occurrence of each sequence and capture the bias in the library (13). A fifth order Markov model outputs the probability of sixth nucleotide given the previous five nucleotides. For example, the probability of a sequence ACTACxxxACGC can be calculated as: probability of ACTAC * probability of x given ACTAC * probability of x given CTACx * probability of x given TACxx * probability of A given ACxxx * probability of C given CxxxA * probability of G given xxxAC * probability of C given xxACG. Note that probability of x given any sequence is equals to 1. Multiplying this probability to the total counts or reads of the sample (for which normalization has to be done) outputs the expected number of occurrence (counts) of the gapped *k*-mer sequence.

Next, the most enriched gapped *k*-mer sequence was used as a seed to generate position weight matrix (PWM) by first calculating enrichment for all gapped *k*-mer sequences having 1-mismatch to the seed sequence in the same manner and then weighting each position of PWM on the basis of the calculated enrichment values (14).

### Library preparation and cloning

The promoter libraries were based on a strong constitutive *E. coli* promoter, apFab71. The promoter libraries were flanked by 3′ and 5′ BsaI digestion sites and a ribosome binding site (Bujard RBS) and were ordered as single-stranded DNA oligos from Integrated DNA Technologies, Inc. To render the libraries double-stranded, we used a primer complimentary to the 3′ of the single-stranded libraries for DNA hybridization and DNA polymerization (reaction: 10 μM library, 10μM primer, 1U Kapa DNA polymerase, 1X Kapa HF buffer, 200 μM dNTPs; protocol: 95°C for 3 min, 60°C for 1 min, 72°C for 3 min). Hybridization products were purified (Omega Bio-tek, Inc.) and the amplicon sizes were verified on a 2% agarose gel. We used Golden Gate Assembly to clone the promoters into designed vector pXL-3 (modified from pJ251-Gerc, Addgene.org plasmid #47441, Supplementary Table S3) to drive the expression of super-folder GFP. We combined pXL-3 backbone with individual promoter library (molar ratio of the backbone and each promoter library was 1:9) and incubated total 300 ng DNA in a 20 ul Gibson Assembly (15) reaction mix at 37°C for 1 h and 60°C for 5 min. We dialyzed Gibson Assembly product on a membrane (0.02 μm pores) in dH_2_O for 1 h to remove salt. We transformed 30 ng DNA from each dialyzed reaction into DH10B electrocompetent *E. coli* (New England Biolabs, Inc.) and recovered at 37°C for 1 h. After recovery, serial dilutions of transformants on LB (50 μg/ml kanamycin) plates yielded ∼500 000 CFUs per transformation.

### Enrichment of functional promoters by fluorescence-activated cell sorting (FACS)

We cloned aTFs into a pSC101 backbone (carrying spectinomycin resistance gene, Supplementary Table S3) under the control of a constitutive promoter and strong RBS by Gibson assembly (15). Assembly reaction was transformed into DH10B electrocompetent cells and allowed to grow overnight at 37°C. After sequence validation, we made cells carrying aTFs electrocompetent by standard procedures and transformed the cells with *in vitro enriched* promoter library. We plated serial dilutions of each transformation and estimated ∼500 000 CFUs per transformation. We made two replicates of cells carrying libraries with aTF and induced one of them with appropriate small molecule inducer. We induced the PmeR library with 250 μM phloretin, TtgR library with 500 μM Naringenin and NalC library with 20 μM PCP. After growing with shaking at 37°C for 5 h, cells were prepared in PBS for flow cytometry (Sony SH800S, Sony Biotechnology, Inc.).

We set up equally spaced fluorescence bin boundaries at 50% PMT and 60% PMT on GFP and mCherry respectively in a cell sorter (SH800S, Sony Biotechnology Inc.). We removed doublets and cell debris by setting forward scattering threshold to 2500 arbitrary units. We gated out doublets by plotting forward scattering area versus forward scattering height and removing events with roughly doubled time (time is a function of area and height in the forward scattering measurement). We sorted 500 000 events per bin and recovered sorted population in 4 ml LB shaking at 37°C for 1 h. Then, we added antibiotics (50 μg/ml kanamycin and 50 μg/ml spectinomycin) and allowed cells to grow to OD_600_ of 0.6. We reinoculated cells from each bin at 1:15 dilution into two replicates and induced one of the replicates with appropriate small molecule inducer. All cultures grew for 5 h before population GFP fluorescence distribution being recorded with the cell sorter.

### Measurement of aTF response curves from individual clones

We randomly selected around 100 colonies from bins with highest fold induction (bins 2 and 3 for PmeR; bin 6 for TtgR; bin 6 for NalC). We inoculated colonies in LB (50 μg/ml Kanamycin and 50 μg/ml Spectinomycin) in 96-well plates and allowed cells to grow until OD_600_ of 0.6. Then, we reinoculated cells in fresh LB at 1:15 dilution either with or without respective inducers. We allowed cells to grow for 5 h before measuring their OD_600_, GFP and mCherry fluorescence on a multi-plate reader (Biotek HTX). We calculated fold induction by dividing induced normalized fluorescence against non-induced normalized fluorescence. We ranked colonies by their normalized induced fluorescence and selected 96 colonies from each aTF initial colony pool with high fold inductions along the normalized induced fluorescence scale. We sequenced 96 colonies from each aTF pool to remove any duplications and sequences with imperfect constant regions on the promoter, RBS and reporter gene *gfp*. At the end, we had 30–60 unique sequences for each aTF (Figure 4 and Supplementary Table S1). We measured induction response for all synthetic and native promoters in three biological replicates with or without appropriate small molecule inducer. We selected 20 representative sequences from each aTF pool to measure inducer dose response (Figure 4C and Supplementary Table S1). We calculated the transfer function parameters using *minpack.lm* package in R (Figure 4D, Supplementary Figure S5). Fitted curves were manually inspected (Supplementary Figures S6–S8).

### Model building with machine learning

For each of the functional operator sequences corresponding to the three prokaryotic transcription factors—PmeR, TtgR and NalC, fold induction ratios were computed based on experimentally measured induced and repressed level responses. As the operator sequences were of varying length, multiple sequence alignment was performed using T-coffee as it yielded the most compact gap-filled sequence representation (16) (Supplementary Table S4). These gap-filled sequences were later converted into numerical feature vectors using one-hot encoding (17). Support vector regression with a radial basis function was used to build quantitative models to accurately predict fold induction ratios for a given operator sequence under 5-fold cross-validation. Additionally, 10% of the initial data-set was held-out to assess the generalizability. The Python based package—scikit-learn v.0.17.1 (17) was used for implementing support vector regression. The scripts were optimized and executed in parallel across 23 CPUs (Intel Xeon 2.4 GHz processors) with cache size capped at 20 GB of RAM space to achieve a computational run time of ∼ O(1h).

### Feature importance analysis

In order to assess which nucleotides at a given position are important, feature importance scores were computed based on the number of times a particular feature was selected among the best set of features obtained at the end of simulated annealing across 100 bootstrap samples of the training dataset, as well as the observed distribution of the feature values among the inducible and non-inducible sets. These feature importance scores were internally normalized and custom position weight matrices were computed for inducible and non-inducible sets for the three different aTFs. Mathematically,

$$\begin{eqnarray*}{\rm{feature}}\_{\rm{importance}}\_{\rm{scor}}{{\rm{e}}_{{\rm{i,j,k}}}} &=& {\rm{bagged}}\_{\rm{frequenc}}{{\rm{y}}_{{\rm{i,j}}}}\nonumber\\ &&\times{\rm{observed}}\_{\rm{frequenc}}{{\rm{y}}_{{\rm{i,j,k}}}}\end{eqnarray*}$$

wherein ‘i’ stands for position number which ranges from 1 to length of gap-filled sequence, ‘j’ stands for sequence character in the set {‘A’,‘T’,‘G’,‘C’,‘-’} and ‘k’ stands for either inducible or non-inducible set, ‘bagged_frequency’ stands for normalized frequency aggregated across 100 bootstrap sampled datasets. Later, sequence logos were generated using the web3logo tool (18).

## RESULTS

### Enrichment of new operator sites by *in vitro* selection

We chose a strong, constitutive, minimal *E. coli* promoter (apFab71) with canonical –35 (TTGACA) and –10 (TATAAT) sites as the starting promoter for design. We call this the ‘template’ promoter because all engineered promoter variants are derivatives of apFab71. We created a library of operators with theoretical diversity of approximately 10^12^ sequences by randomizing the spacer bases between –35 and –10 sites. Although spacers in natural promoters are typically 17 bp long, we created 16, 17, 18 and 19 bp long spacer libraries to provide greater adaptability for binding to an aTF. Prior to *in vitro* selection, we validated that all three aTFs indeed bind and release their native binding site in a ligand-dependent manner by gel shift measurements (Supplementary Figure S2 and Supplementary Table S2). Then, we assessed the impact of randomizing the spacer on constitutive promoter activity (aTF not co-expressed) by comparing GFP expression of the unselected promoter library with unmodified apFab71. Promoter libraries from 16, 17 and 18 bp spacers were highly active (aTF not co-expressed) with median fluorescence distribution of population comparable to unmodified apFab71 promoter (Supplementary Figure S1A and B). However, the 19 bp promoter library was less active, which was likely caused by misalignment of –35 and –10 sites weakening RNA polymerase binding (Supplementary Figure S1B). Nonetheless, we included the 19bp promoter library because a longer operator sequence may be more suitable for aTFs with a larger binding footprint. We chose three aTFs of the TetR-like family: PmeR and TtgR bind to different flavonoid molecules which are natural products used in medicinal, nutraceutical and cosmetic applications, and NalC binds to pentachlorophenol which is an environmental toxin used as herbicides and wood preservatives against fungal infection. We carried out *in vitro* selection by overexpressing His-tagged aTF as bait in *E. coli* cell lysate. Equimolar amounts of all four promoter libraries were mixed and incubated with the cell lysate, followed by protein pulldown and PCR amplification of bound sequences. Deep sequencing after five rounds of selection gave an estimated 10^5^ unique operators for each aTF (Supplementary Table S4).

**Figure 2.**
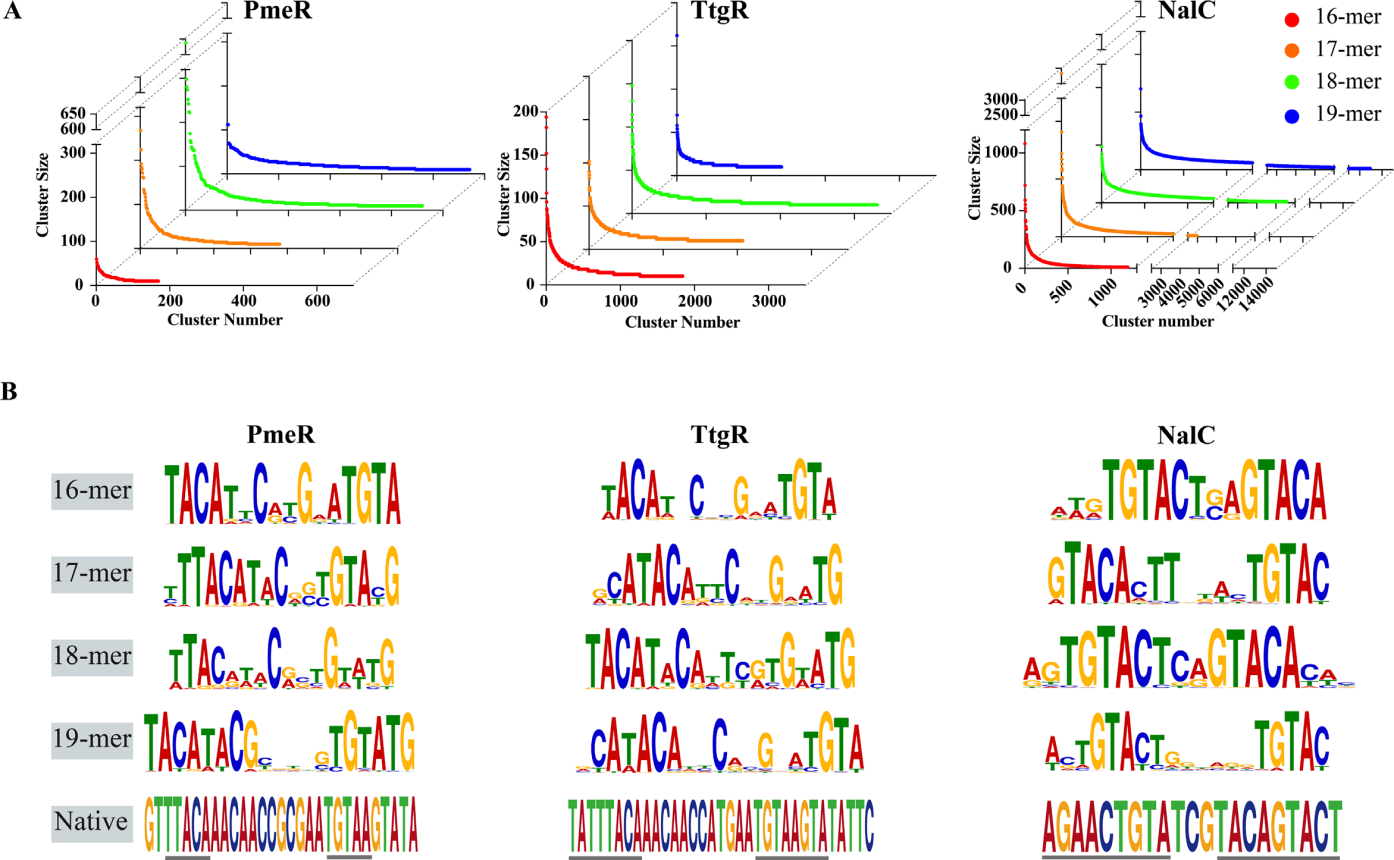
*In vitro* selection of operators. (**A**) Highly similar operators are clustered together at 90% sequence identity threshold. Clusters ranked in descending order (left to right) by number of sequences within a cluster or cluster size (Y-axis). Cluster size shows characteristic exponential fit. Minimum number of sequences per cluster is five. Red, orange, green and blue represent operator libraries of length 16bp, 17bp, 18bp and 19bp, respectively. (**B**) Motifs of highly enriched sequences of all four operator libraries and native operator sites of PmeR, TtgR and NalC. Palindromic sequences representing putative half sites is underlined in native operator sites.

To assess the results of *in vitro* selection, we eliminated sequences with fewer than 10X coverage, clustered the remaining sequences at 90% sequence identity threshold (using CD-HIT (19)), and sorted the clusters in descending order based on their size (i.e., number of sequences within each cluster) for each of the promoter libraries individually. The ordered list of clusters for all three aTFs gave a characteristic exponential fit indicative of successful enrichment as seen in other SELEX-based methods (20,21) (Figure 2A). Because sequence enrichment was broadly distributed, we inferred that the selected library contains operators with different affinities for the aTF, which is an important requirement for our design strategy to generate varying transcriptional outputs. No-aTF negative control did not show exponential ranking of cluster sizes as expected. For each aTF, similar binding motifs emerged within the respective N-mer libraries indicating convergence of *in vitro* selection (Figure 2B). No-aTF control gave random, low information content motifs consistent with poor enrichment (Supplementary Figure S3). Strong palindromic signature was evident among the core motifs validated that PmeR, TtgR and NalC function as dimers as expected with each half site 4–6 bp long and separated by 2–6 bp (Figure 2B).

A striking observation is that *in vitro* selection has radically reshaped operator site configuration compared to the native operator while retaining core binding motifs of the latter. Native binding sequence for PmeR (22) and TtgR (23) are nearly 30bp long with binding site for each monomer (half site) of the dimer separated by 12–13 bp (Figure 2B). Yet, we found that the core binding motif of PmeR and TtgR could be accommodated within our much shorter 16–19 bp operator libraries with only 6-8bp separating the half sites (Figure 2B). There are two plausible structural interpretations: (i) any local structure between half sites such as bending of DNA that may be present in the native promoter no longer exists in *in vitro* operators (ii) crossing angle between the DNA-binding domains of individual monomers could be greater with native binding site, but narrower with *in vitro* operators. Interestingly, we observe the effect in NalC where separation between half sites is longer in the *in vitro* operator library (6 bp) compared to the native binding site (24) (3 bp) (Figure 2B). Taken together, these results suggest that aTF-promoter interactions have a high degree of structural plasticity while preserving function. In summary, *in vitro* selection is a simple, yet powerful approach to generate operators with a wide range of aTF binding affinities, allowing aTFs to access new operator configurations distinct from their native binding site.

### Identification of functional promoters by cell sorting

**Figure 3.**
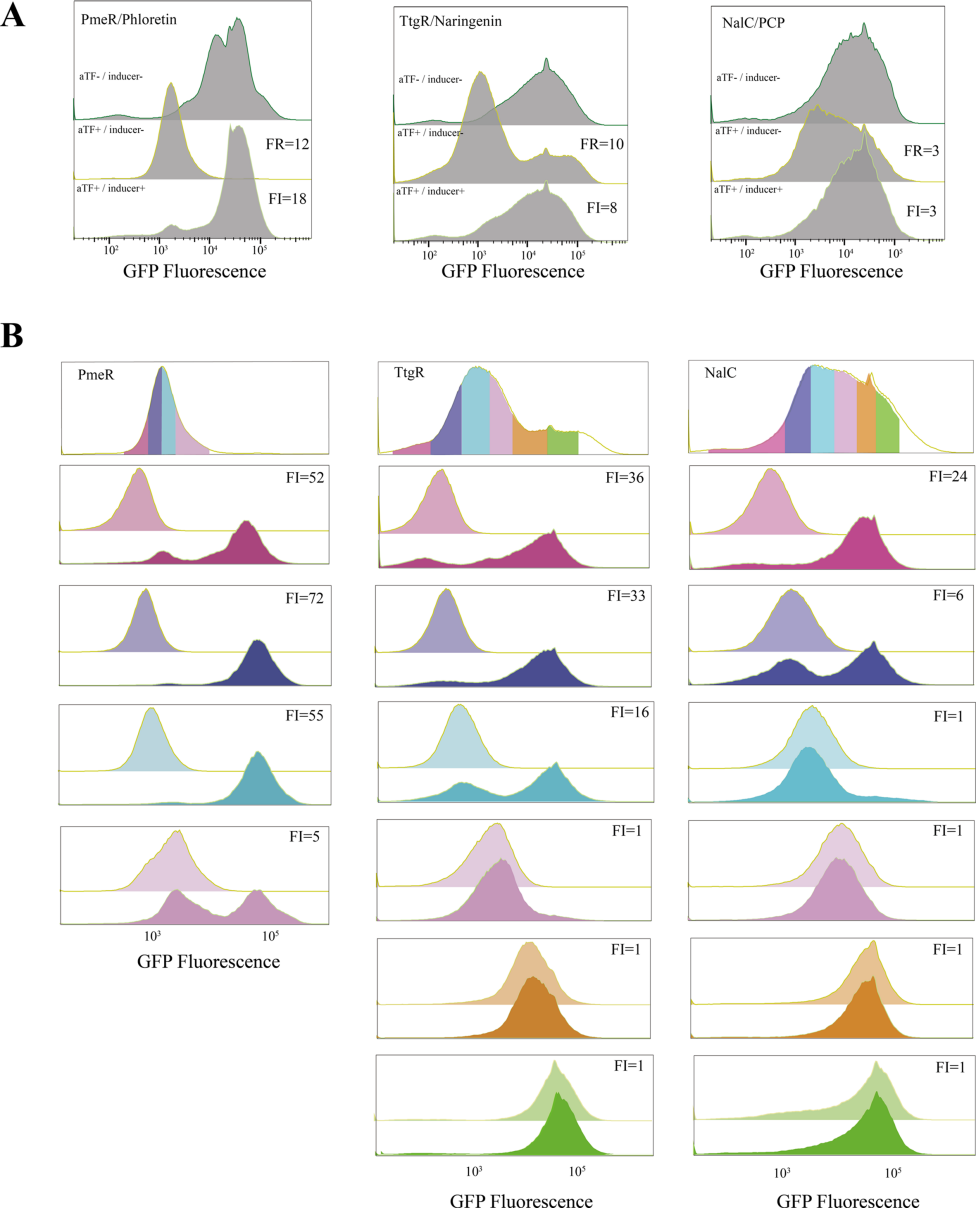
*In vivo* enrichment of inducible promoters by fluorescence-activated cell sorting. (**A**) Cells expressing GFP regulated by engineered promoter variants constitu-tively (top, aTF-/inducer-), co-expressing aTF without inducer (middle, aTF+/inducer-), and co-expressing aTF with inducer (bottom, aTF+/inducer+). Fold repression (FR) is the ratio of median fluorescence of aTF-/inducer- and aTF+/inducer- cells. FR is shown in the middle panel. Fold induction (FI) is the ratio of median fluorescence of aTF+/inducer+ and aTF+/inducer- cells. FI is shown in the bottom panel (**B**) Cells repressed by aTF (aTF+/inducer-) are sorted into bins according to their fluorescence and each bin is induced independently. Top panel is overall distribution of repressed cells. Colors represent cells sorted into different fluorescence bins. Lower panels show ligand-induced response of cells from each bin. Fold induction (FI) ratio of each bin is mentioned in the panel.

We employed fluorescence-activated cell sorting to rapidly enrich functional or inducible promoters. Promoter libraries embedded with *in vitro* selected operators for PmeR, TtgR and NalC were cloned upstream of GFP, and transformed into *E. coli*. Transformed cells lacking aTF (aTF–) gave high fluorescence confirming that promoters are still constitutively active (Figure 3A, top). Cells carrying the promoter library and co-expressing an aTF without inducer (aTF+/inducer-) gave markedly reduced fluorescence with a fold change (ratio of median fluorescence of aTF– and aTF+/inducer-cells) of 10.4-, 12.4- and 2.5-fold for TtgR, PmeR and NalC, respectively (Figure 3A, middle). This validated our hypothesis that *in vitro* selected operators embedded between –35 and –10 sites should be able to repress transcription. Fluorescence distribution of the repressed population (aTF+/inducer-) spanned 50–100-fold indicating that the operator affinity for aTF varies widely across the promoter library. Fluorescence distribution of repressed TtgR promoter library (aTF+/inducer-) is distinctly bimodal with a small fraction of high GFP cells which likely contain operators with low or no affinity for TtgR (Figure 3A, middle).

Next, we assessed inducibility of the promoter libraries by determining fold induction upon adding ligand (aTF+/inducer+) below toxicity threshold to repressed cells (Supplementary Figure S4). Fold induction is the ratio of median fluorescence of induced (aTF+/inducer+) and repressed (aTF+/inducer-) cells. Indeed, all three promoter libraries were ligand-inducible, with 17.6-, 7.7- and 2.5-fold induction for PmeR, TtgR and NalC, respectively (Figure 3A, bottom). We then sorted repressed cells from low-to-high fluorescence bins and subsequently induced cells from each bin independently (Figure 3B, Supplementary Table S5). This strategy of slicing the entire population into bins facilitates rapid identification of subpopulations of cells containing inducible promoters over those that are constitutively active. This is best exemplified with NalC where fold induction of the overall population was a modest 2.5-fold, but after cell sorting the purple and blue bins gave fold induction ratios of 24.2 and 6.5, suggesting that these bins are now substantially enriched with inducible promoters (Figure 3B). A high proportion of promoters in the remaining four bins (cyan, pink, brown and green) are constitutively active (fold induction ∼1.0) likely due to low aTF-operator affinity. Cells from three low-fluorescence TtgR bins (purple, blue and cyan) and all four PmeR bins were enriched in inducible promoters with fold induction >5.0 for each bin (Figure 3B). Notably, a small but significant fraction of cells from low-fluorescence bins remain uninducible, and these likely represent operators with very high affinity for aTF.

### Characterizing promoter activity and response function

**Figure 4.**
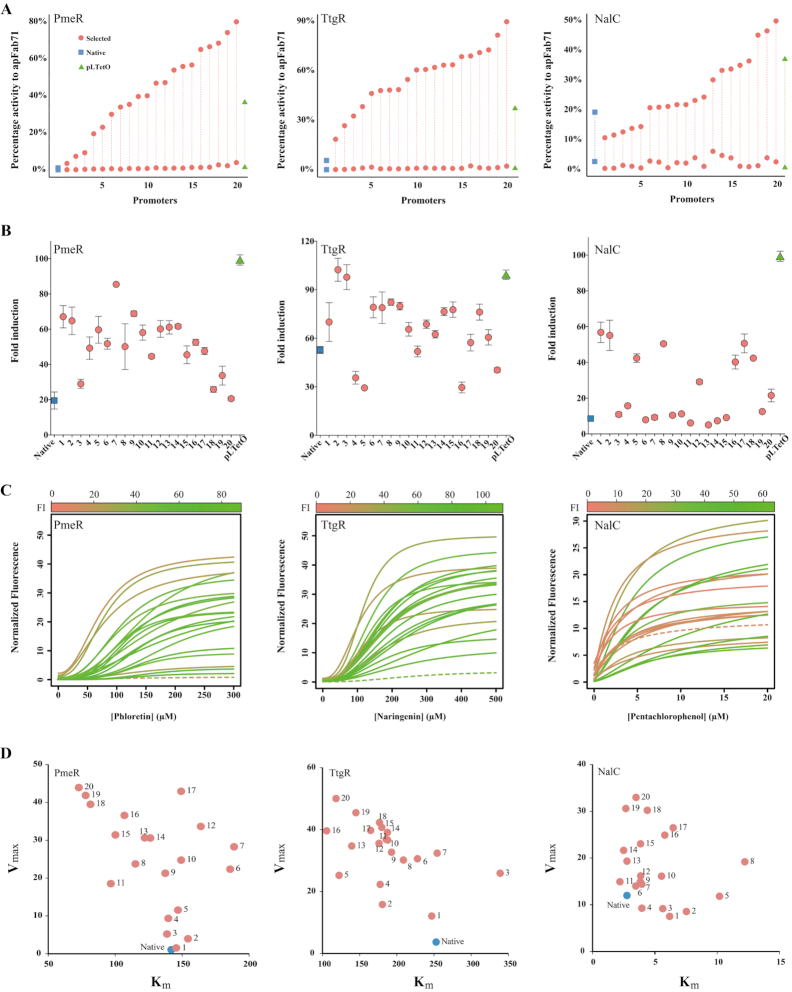
Characterization of transcriptional activity of individual promoters. (**A**) Normalized fluorescence of uninduced and induced states (maroon circles) reported as a percentage activity of constitutive apFab71 promoter. Activity of native promoter ported into *E. coli* (blue squares) and commonly used pLTetO promoter (green triangles) are shown for comparison. (**B**) Fold induction ratio between induced and uninduced fluorescence of engineered, native and pLTetO promoters. (**C**) Ligand dose response data fitted to a standard Hill equation. Color gradient represents fold induction and native promoter is shown as dashed line. (**D**) Plot of maximum induced reporter expression (*V*_max_) versus concentration of ligand required to reach half *V*_max_ (*K*_m_). Both parameters estimated from fitted Hill equation. Blue dot represents native promoter.

For each aTF, we clonally evaluated promoters by testing ∼100 randomly selected colonies from each sorted subpopulation with high fold induction to find 30–50 inducible promoters. Selected promoters for all three aTFs gave a broad range of gene expression properties in terms of maximum reporter expression and dynamic range of the reporter signal (ratio of induced to uninduced reporter expression) (Figure 4A and B, Supplementary Table S1). All promoters had low induced baseline expression or a tight off state but different levels of ligand-induced expression (Figure 4A). Maximum ligand-induced expression across promoters ranges as gradual increments from 3–80% (PmeR), 20–90% (TtgR) and 10–50% (NalC) of constitutive apFab71 (original template promoter) which is the upper limit of gene expression (Figure 4A). Strongest induced promoter of PmeR, TtgR and NalC is 77-, 16- and 66-fold more active over their respective native promoters ported into *E. coli* (Blue points, Figure 4A). Furthermore, strongest induced promoter of PmeR and TtgR was greater than twice as active and NalC 40% more active than the widely used pLTetO promoter (25) (Figure 4A, green points). Fold induction of individual promoters (ratio of fluorescence of induced to uninduced states), which denotes dynamic range of reporter signal, of all twenty PmeR and NalC promoters and sixteen out of twenty TtgR promoters was greater than their respective native counterparts (Figure 4B). Although the dynamic range of native promoters of TtgR and PmeR was high (55- and 20-fold, respectively, blue squares) owing to low uninduced baseline expression, their overall activity was poor. But, engineered promoter variants with comparable uninduced baseline expression gave much higher dynamic ranges due to greater transcriptional activity (Figure 4B). This difference is best exemplified by comparing native promoters of PmeR, TtgR and NalC with respective engineered variants of comparable dynamic range (PmeR variant 20, TtgR variant 20 and NalC variant 19) (Figure 4B). Although the dynamic range of each pair is comparable (Figure 4B), engineered promoter variants are 50 to 100-fold more active (Figure 4A). Low activity of native promoters in *E. coli* indicates poor cross-host compatibility and underscores a major challenge in the biosensor field of limited portability of regulatory parts across hosts. By using a template promoter from the target host itself (in this case, *E. coli*), we simplify aTF portability and achieve high activity.

Next, we measured ligand concentration dependent gene expression (dose response) of all promoters and fitted the data to a standard Hill equation. The dose response curves revealed rich functional diversity of promoter variants with different ligand sensitivities and transcriptional activities compared to native promoter ported in *E. coli* (Figure 4C, solid lines versus dashed line). To quantify the response characteristics, we computed the following Hill equation parameters *V*_max_, *K*_m_ and *n* by non-linear regression (Supplementary Table S1, Supplementary Figure S6–S8). *V*_max_ is the maximum promoter activity at full induction; *K*_m_ is the concentration of ligand for half *V*_max_ expression and represents sensitivity to the ligand; and *n* is the Hill coefficient describing cooperativity of response. Several interesting properties of the promoters become evident from inspecting Hill equation parameters (Figure 4D). First, points parallel to the X-axis (Figure 4D) are promoters with different ligand sensitivities (*K*_m_) but similar gene expression levels (*V*_max_). The widest gap in *K*_m_ values for similar *V*_max_ are the following promoter pairs: PmeR promoters 20 versus 17 (77 μM versus 149 μM), TtgR promoters 5 versus 3 (122 μM versus 339 μM), and NalC promoters 8 versus 11 (2.8 μM versus 12.2 μM). This result is non-intuitive because it shows ligand sensitivity can be altered by changing operators without mutating the protein itself. One may expect ligand sensitivity to be linked to ligand affinity or allosteric activity which are properties of the aTF, not the operator. Second, changing operators can change cooperativity of signal response (Supplementary Figure S5). Greater cooperativity (higher Hill coefficient) implies both monomers of the dimeric aTF act in concert eliciting a steeper response and lower cooperativity generates a graded linear response (Supplementary Figure S5–S8). Since cells utilize both types of signal responses, our results show that natural promoters may adapt signal response to different contexts by possibly changing operators. These results open the possibility of a whole new layer of genome regulation where transcriptional response is encoded in the operator and is reminiscent of studies demonstrating alternate ligand specificities of glucocorticoid receptor at different operator sites (26,27). Third, promoters with similar *K*_m_ have different *V*_max_ (lines parallel to Y-axis, Figure 4D). Since *in vitro* selection generates operators with different affinities, an operator with greater aTF affinity would have lower *V*_max_ due to higher residual binding even upon induction compared to one with lower affinity. In summary, characterization of individual promoters shows that a multitude of induction responses can be generated by simply altering operator sequences.

### Sequence determinants of promoter function by machine learning

**Figure 5.**
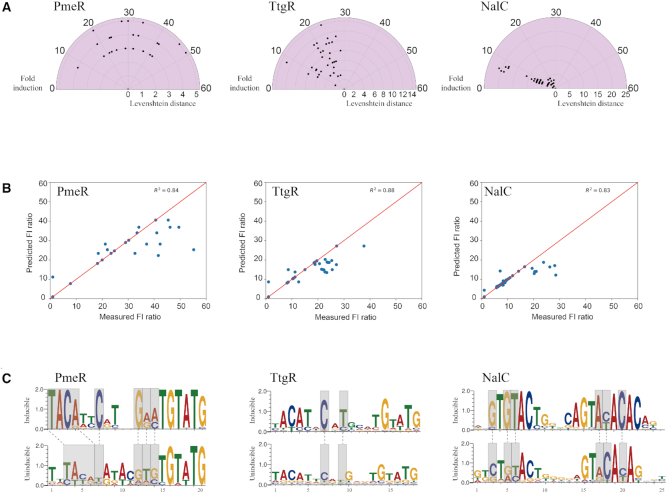
Sequence determinants of promoter activity by machine learning. (**A**) Diversity of operator sequences and their corresponding fold induction shown as a speedometer plot. Central point corresponds to a reference sequence, in this case operator embedded with highest fold induction. Radial axis is Levenshtein distance between reference operator (center point) and the remaining inducible sequences. Angular axis is fold induction. (**B**) Scatter plots of fold induction predicted vs. experimental and the Spearman correlation coefficient. (**C**) Sequence motifs of inducible (top) and uninducible (bottom) operators. Y-axis represents bits. Gray boxes indicate differences in key sequence features between inducible and uninducible promoters. Logos were generated using web3logo tool

Since only a subset of promoters that repressed the reporter were ligand-inducible and the rest were constitutively off, we wanted to understand the sequence determinants of promoter function. We hypothesized that both inducible and uninducible promoters likely have the same core motif required for binding, but position of the core motif and/or identity of the flanking bases may play an important role in ligand inducibility. To test this hypothesis, we developed machine learning models for each aTF independently using inducible and uninducible sequences, corresponding to the top one percent of most abundant sequences from *in vitro* binding assay, to assess the contribution of different nucleotides at a given position. It is well known that sampling sequence space uniformly helps improve predictive power of empirical models for making predictions on unexplored regions. To retrospectively assess sequence and functional diversity in our dataset, we plotted edit (or Levenshtein) distance between sequence of interest and a reference sequence (along the radial axis) vs. fold induction ratio (along the angular axis) (Figure 5A). While PmeR and TtgR sequence variants were broadly distributed, sequence diversity of NalC was relatively lower. As the operator sequences were of varying length (16–19 bp long), we performed multiple sequence alignment using T-coffee to yield the most compact gap-filled sequence representation (16) (Supplementary Table S6). Based on one-hot encoding, each sequence was converted into a vector of numerical features corresponding to the identity of bases at a given position (Supplementary Figure S9). We used support vector regression (28) with radial basis function to build quantitative models to accurately predict fold induction ratios for a given operator sequence. The coefficient of correlation between predicted and actual fold induction ratios was 0.84, 0.88 and 0.83 for TtgR, PmeR and NalC, respectively (Figure 5B, Supplementary Table S7). In order to assess which nucleotides at a given position are important, we computed feature importance scores based on the number of times a particular feature was selected at the end of simulated annealing across 100 bootstrap samples of the training dataset. Next, we identified consensus sequence motifs (18) for operators within inducible and uninducible promoters based on the feature importance scores computed by machine learning. Both inducible and uninducible promoters share similar core motifs resembling the motifs obtained after *in vitro* selection (Figure 2) which suggests that these motifs are minimally required to bind to aTF (Figure 5C). Differences between inducible and uninducible promoters arise from location of the core motif within the operator region, as well as the identity of bases flanking the core motif. For PmeR, ‘TACA’ core motif of the left half site is located between nucleotides 1–4 among inducible promoters and 3–6 among uninducible promoters. A plausible explanation is that a wider binding angle caused by TACA at positions 1–4 may increase DNA bending or strain the dimer interface and the resulting strain energy may allow PmeR to readily dissociate upon induction. Bases flanking the core motif also help drive the differences between both sets of promoters (Figure 5C). They can be completely different as seen at positions 7 and 13 of PmeR. But more often, they show stronger sequence preference for a certain base(s) as seen at positions 12 and 14 of PmeR, 7 and 9 of TtgR, and 3, 5, 6, 17, 18 and 20 of NalC. Pairwise and higher-order synergistic interactions between bases may also facilitate inducibility of a promoter as simple linear models failed to explain the variability in data. These results show that seemingly minor differences in promoter sequences can have a large impact on inducibility of a promoter. When a ligand binds to a DNA-bound aTF, strain energy propagates from ligand to the DNA-binding domain. Minor sequence differences may control the balance between a tightly bound state that cannot be released and a weakly bound state that can be easily dislodged upon binding of ligand. Since many operons with different transcriptional demands are often controlled by the same aTF, our results provide insights into how nature might tailor the operator site to re-use the aTF to meet different regulatory needs.

## DISCUSSION

Programmable ligand-inducible promoters are an invaluable tool for synthetic circuit design and biosensor-guided high-throughput screening. We designed inducible promoters by modularized assembly onto the simplest of bacterial promoters, devoid of complex regulation found in native promoters. By simply changing aTF’s affinity for its operators, we engineered promoter variants with different gene expression levels, dynamic ranges and ligand sensitivities. This generalizable method should be applicable to other TetR-family proteins and potentially thousands of other bacterial repressors expanding the suite of aTFs for synthetic biology applications. We inserted the operator between the –35 and –10 sites of an *E. coli* promoter because it mimicked the location of natural operator sites for many aTFs and hence would likely be most effective region for blocking the RNA polymerase. This architecture imposes a limitation on length of the operator because transcriptional strength is highly sensitive to the distance between –35 and –10 sites (optimal separation of ∼17 bp). Our methodology can be easily modified to explore other promoter architectures to accommodate aTFs with longer binding footprint. Length is no longer a limitation if the operator is placed upstream of –35 site, flanking –35 on both sides, flanking –10 on both sides or downstream of –10 site. To improve the efficiency of *in vitro* selection, we can facilitate binding by *a priori* fixing the core motif from natural operators and randomizing the remaining positions. Our promoter engineering strategy should be applicable to transcription activators too because though they are mechanistically different from repressors, the underlying principles governing aTF-operator interactions remain the same. For instance, transcription activators of AraC-, LysR- and XylR-like families translocate across distal and proximal operator sites with differential affinities in a ligand-dependent manner to mask or unmask RNA polymerase binding sites within a promoter (29). Operators with different affinities at the distal and proximal sites would modulate transcription by changing promoter occupancy by RNA polymerase. A systematic study of different architectures and aTF mechanisms would lead to comprehensive design principles for ligand-inducible promoter engineering.

Discovery of new metagenomic aTF biosensors for high-throughput screening would be bolstered by our method (30,31). The metagenome is a treasure trove of new aTF biosensors, but often native promoters are either unknown or cannot be activated. This would no longer be an impediment because we should be able to *de novo* engineer inducible promoters for metagenomic aTFs by our method. Non-model organisms have attracted renewed interest, enabled by CRISPR-Cas9- genome editing, because their novel metabolic capabilities can be harnessed for biosynthetic engineering (32). Although aTF-based biosensors are widely employed in *E. coli*, their use is virtually non-existent in non-model organisms due to poor compatibility of promoters across hosts. Our method should simplify aTF portability into industrially important non-model microbes including *Corynebacterium, Pseudomonas* and *Zymomonas* by using a constitutive promoter of the native host, and adjusting aTF-operator interactions to match the strength of the native promoter.

Our result also challenges the passive role attributed to an operator as merely a docking or tethering site of the aTF. This view arises from perceiving the ligand as the allosteric effector and DNA-binding interface as the distal site. We show that changing the operator can profoundly affect transcriptional response such as ligand sensitivity and cooperativity which are generally thought of as properties of the protein, not DNA. Protein allostery could be potentially bidirectional where both allosteric and distal sites ‘talk’ to each other rather than the conventional unidirectional paradigm. Furthermore, this would expose a whole new layer of site-specific transcription regulation governed by aTF-operator interactions.

## Supplementary Material

gkz772_Supplemental_File
