# Supplementary material for: De novo design of programmable inducible promoters
Source: Nucleic Acids Res. 2019 Sep 25;47(19):10452–63. doi: 10.1093/nar/gkz772 (PMC6821364; doi:10.1093/nar/gkz772)
Supplement: gkz772_Supplemental_File [file gkz772_supplemental_file.pdf]

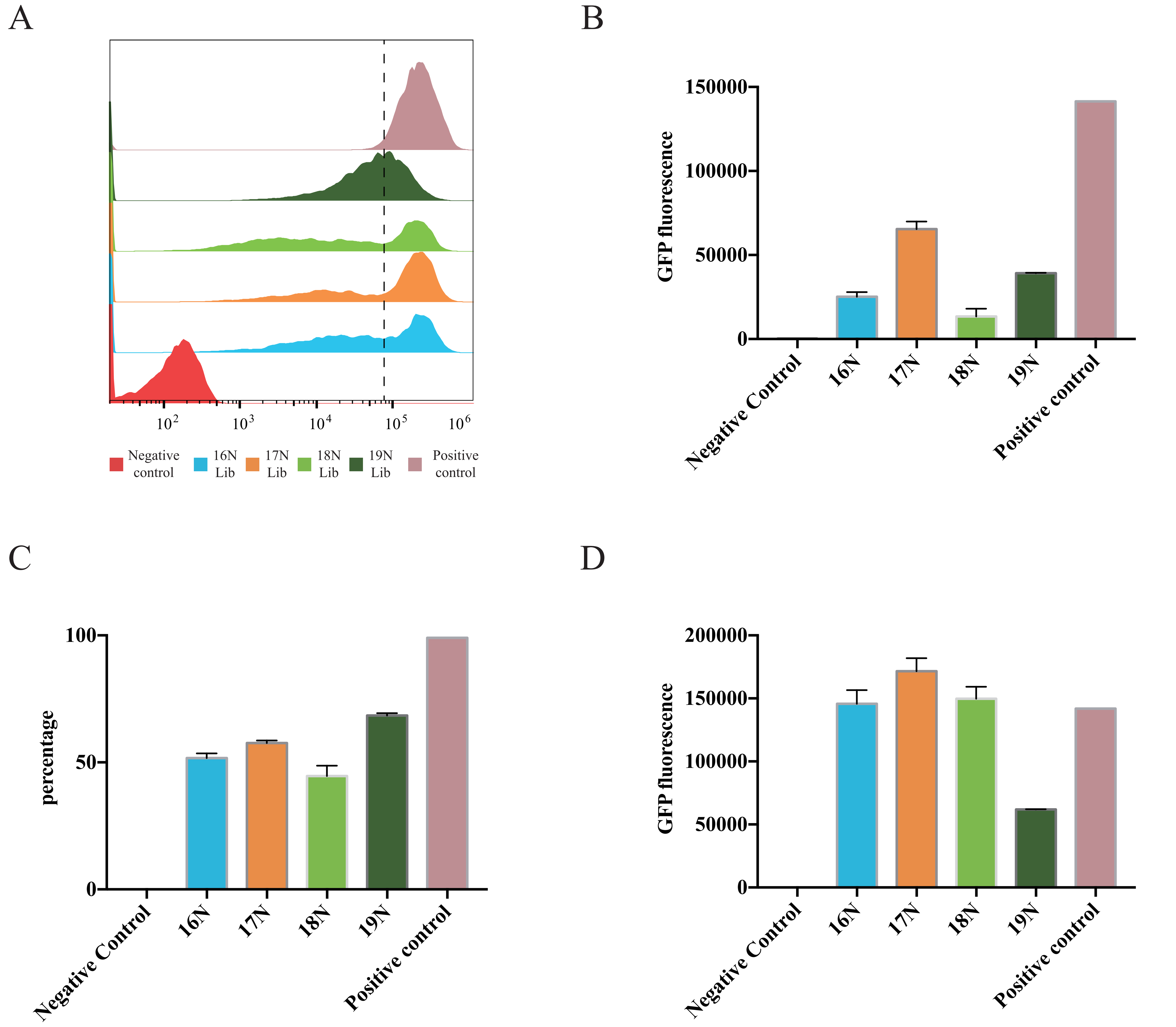

Figure S1: Comparison of gene expression of template promoter apFab71 and promoter library with randomized spacers (A) Flow cytometry distribution of apFab71 (positive control) and promoter variants embedded with randomized 16-, 17-, 18- , 19-mers and the negative control. The negative control was DH10B cells carrying no plasmids. **(B)** Median fluorescence of cells from positive control, 16N library, 17N library, 18N library, 19N library and the negative control. **(C)** The percentage of cells above lower activity threshold of apFab71 (dashed line) (D) Median fluorescence of cells above lower activity threshold of apFab71 (dashed line).

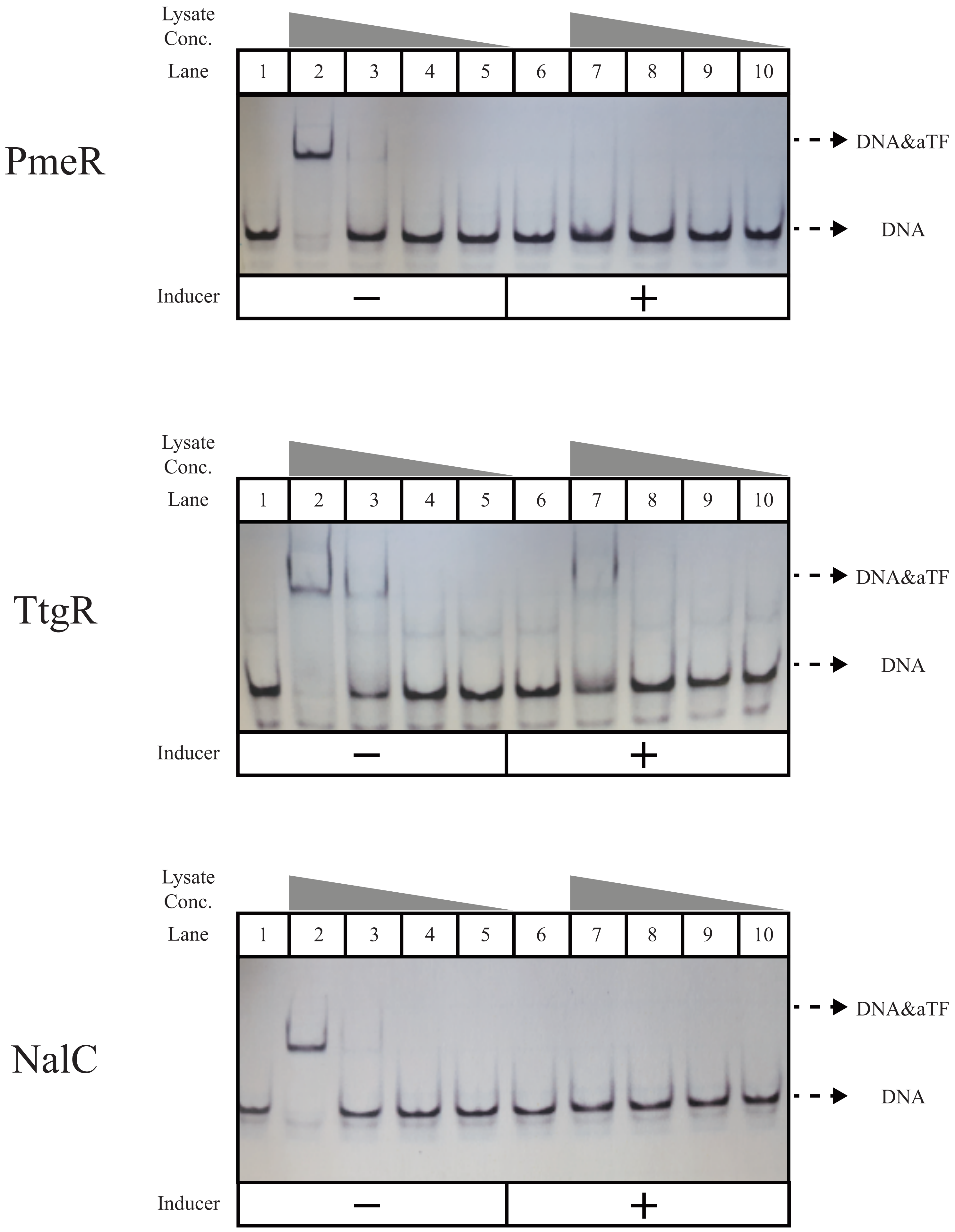

Figure S2: Gel shift assay of PmeR, TtgR and NalC with their respective native binding site. Relative concentration of cell lysate and ligand concentration is indicated. PmeR was induced with 1mM phloretin. TtgR was induced with 1mM naringenin. NalC was induced with 1mM pentachlorophenol. The lysate was diluted 1 time (lane 1, 2, 6 and 7), 5 times (lane 3 and 8), 25 times (lane 4 and 9) and 125 times (lane 5 and10)

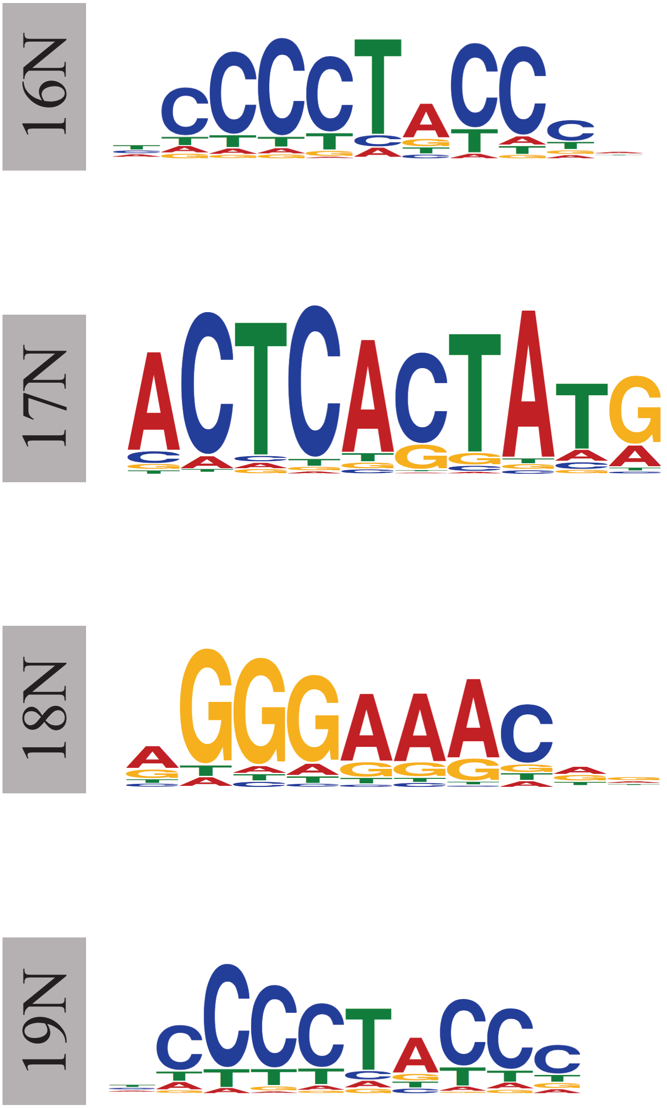

Figure S3: Sequence motifs from no-aTF negative control after *in vitro* selection.

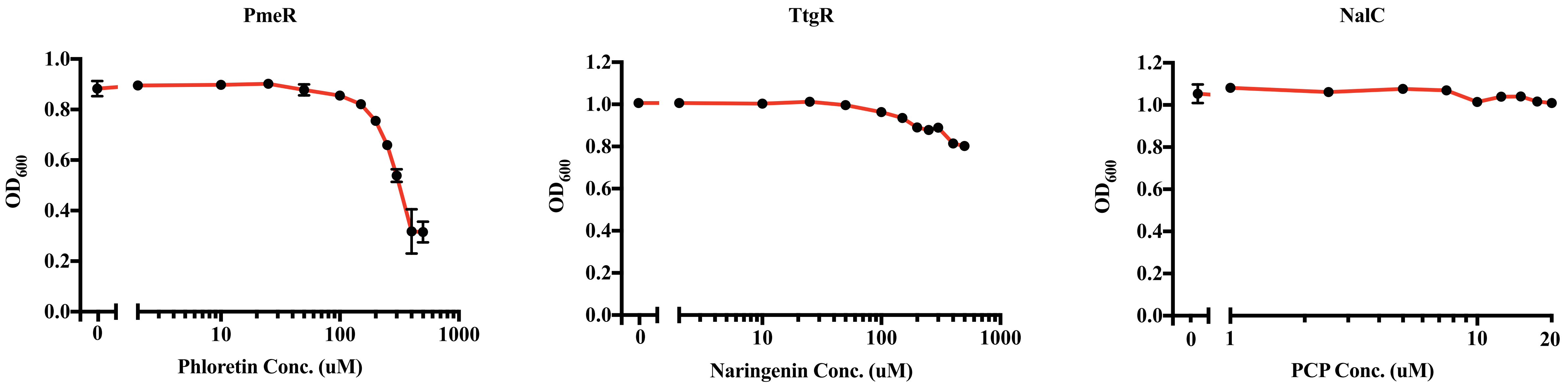

Figure S4: Toxicity of inducer ligands, phloretin, naringenin and pentachlorophenol. Y-axis is absorbance at OD600

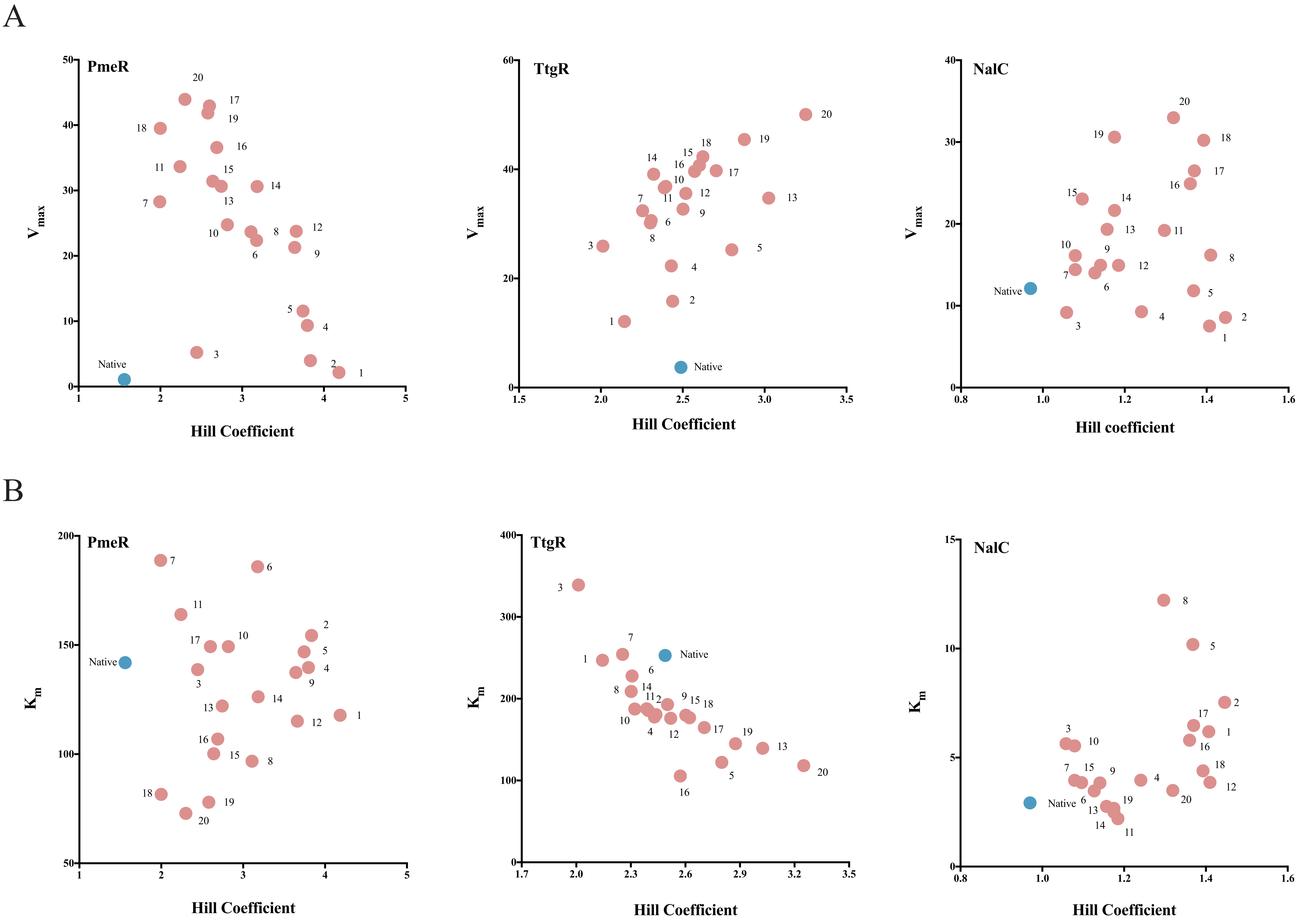

Figure S5: Parameters of transfer functions. (A) Hill coefficient n vs. maximum induced expression Vmax of PmeR, TtgR and NalC. **(B)** Concentration needed to half V_{max} K_m vs. maximum induced expression V_{max} of PmeR, TtgR and NalC.

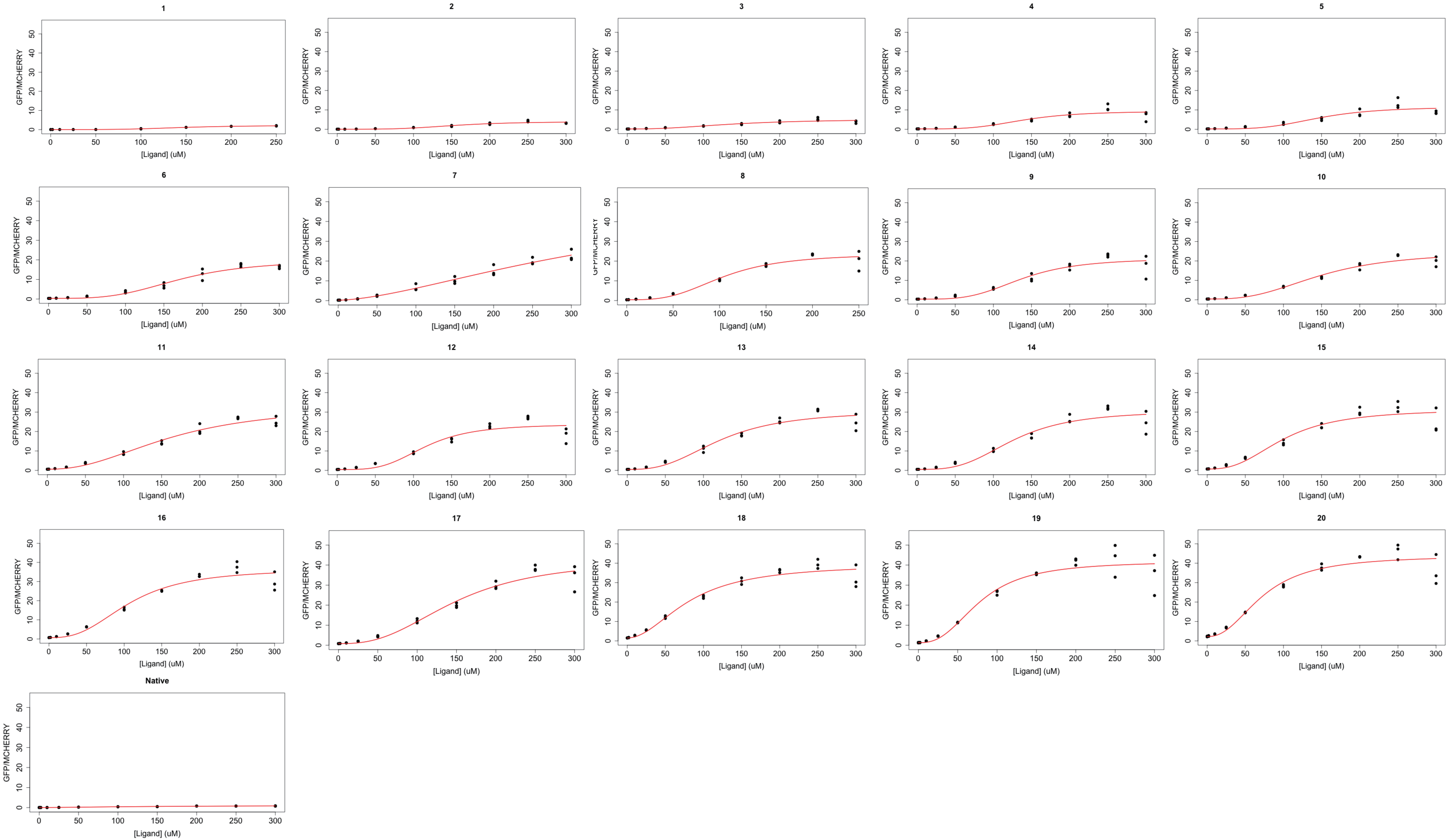

Figure S6: Curve fits of individual promoter variants of PmeR. Mean of three biological replicates at each ligand concentration used for curve fitting. Promoters 1 and 8 were only fitted to 250μM to avoid artificially high hill coefficients from mis-fitting.

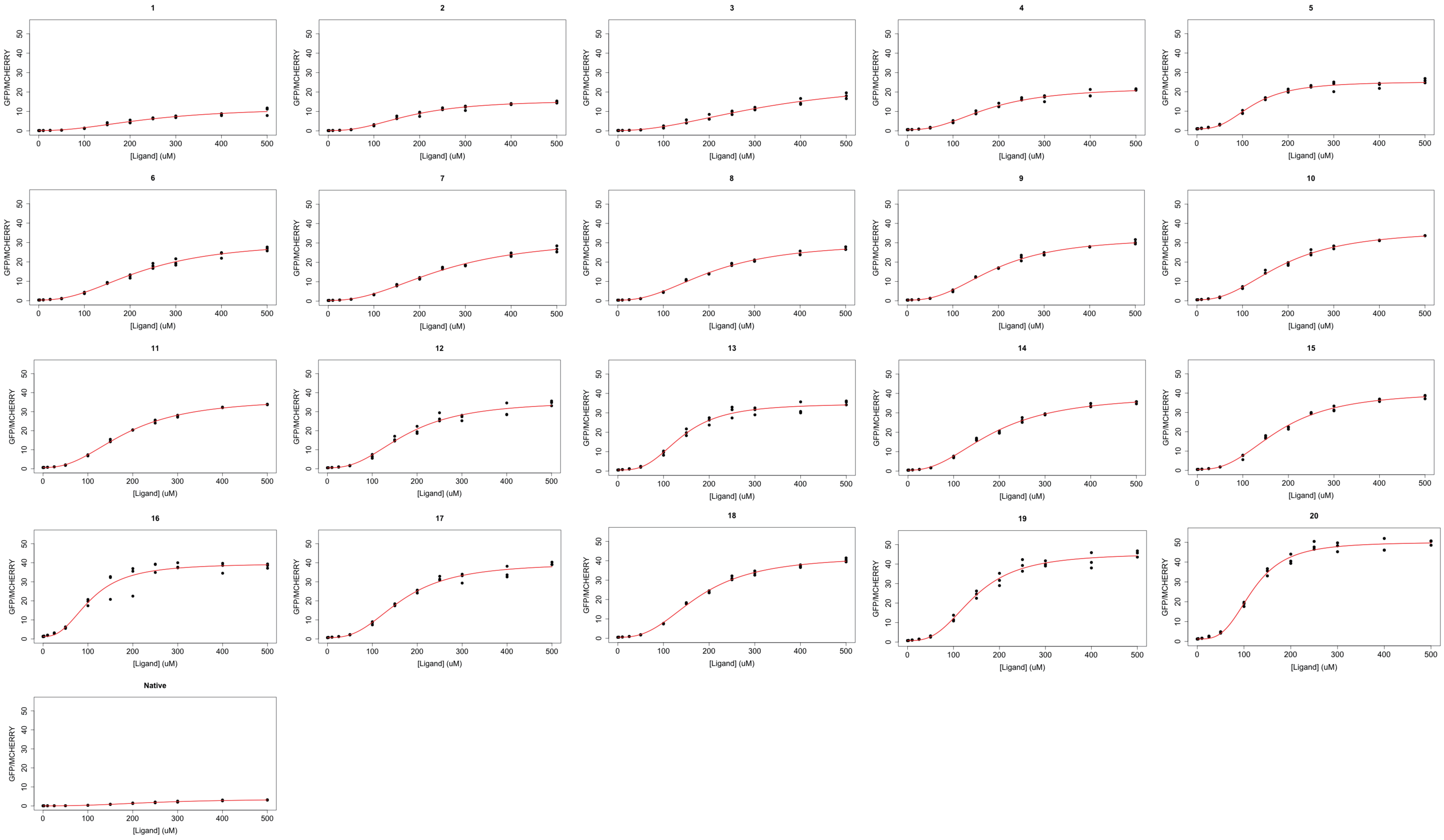

Figure S7: Curve fits of individual promoter variants of TtgR. Mean of three biological replicates at each ligand concentration used for curve fitting.

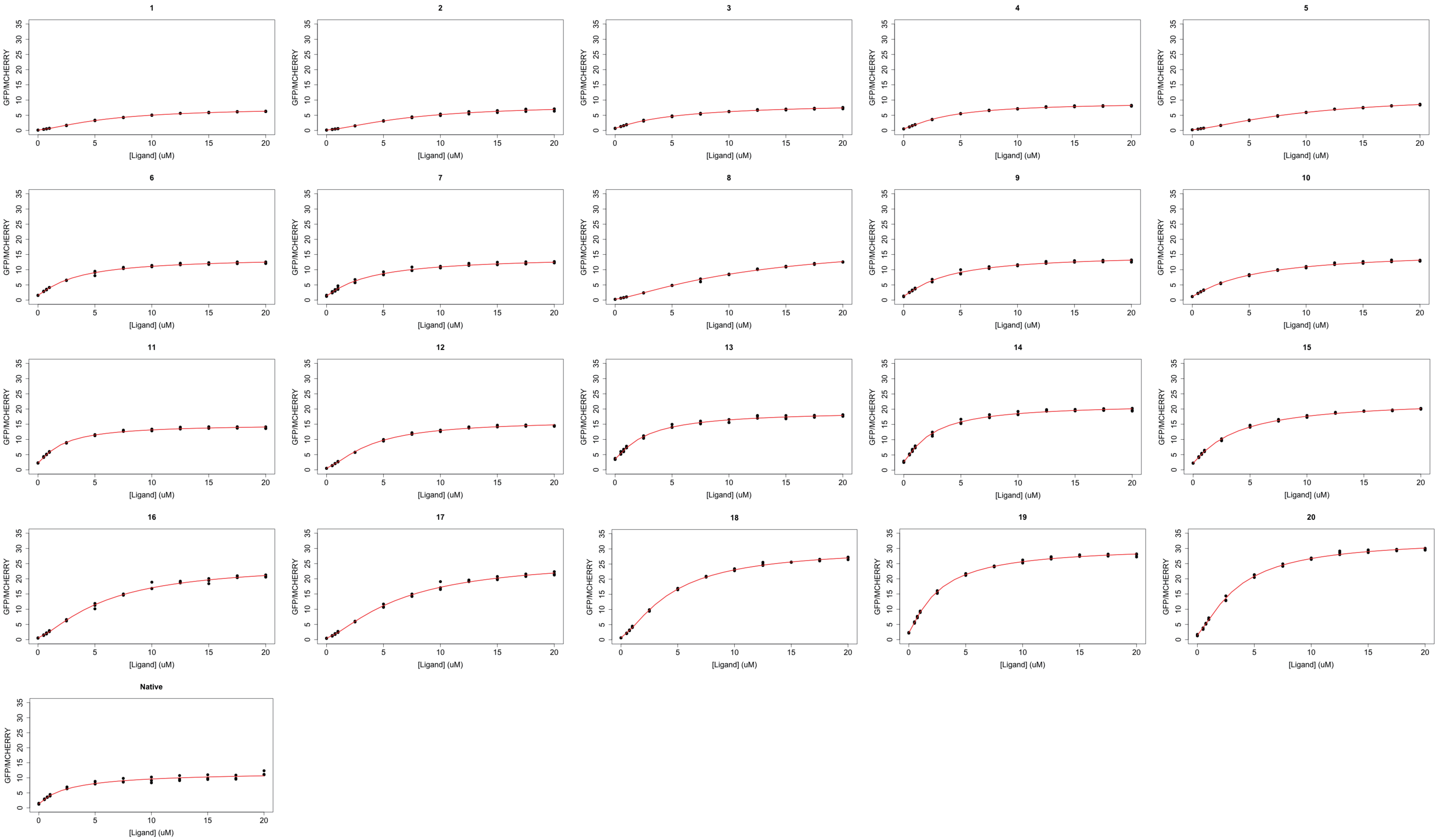

Figure S8: Curve fits of individual promoter variants of NalC. Mean of three biological replicates at each ligand concentration used for curve fitting.

Table S1: Details of sequences used in Figure 4. Repressed=Normalized GFP Fluorescence under repressed state; Induced= Normalized GFP Fluorescence under repressed state; FI=Fold induction; Repressed ratio to apFab71=Repressed GFP fluores--cence divided by that of apFab71; Induced ratio to apFab71=Induced GFP fluores-cence divided by that of apFab71

PmeR

Name	Sequence	Repressed	Induced	FI	Repressed ratio to apFab71	Induced ratio to apFab71
Native	GTTTACAAACAACCGCGAATGTAAGTAT	0.03	0.60	18.68	0.0006	0.0108
1	TACATTCGTGTATGTATG	0.03	1.96	66.82	0.0005	0.0351
2	TACATTCGTGGATGTATG	0.06	4.18	64.36	0.0012	0.0751
3	TACATCCGTGAATGTATG	0.18	5.28	29.08	0.0033	0.0948
4	TACAGTCGTGAATGTATG	0.23	11.15	49.31	0.0041	0.2002
5	TACATTCGTGAATGTATG	0.22	13.27	60.00	0.0040	0.2382
6	TACATTCATGACTGTATG	0.33	17.29	51.70	0.0060	0.3104
7	TACATACATGAATGTTTG	0.23	19.54	85.42	0.0041	0.3508
8	TACATCCATGACTGTATG	0.41	20.39	50.01	0.0073	0.3661
9	TACGTTCATGAATGTATG	0.33	22.82	68.79	0.0060	0.4098
10	TACATGCATGGATGTATG	0.40	22.93	57.81	0.0071	0.4118
11	TACAGCCATGAATGTATG	0.61	27.01	44.55	0.0109	0.4849
12	TACAATCATGGATGTATG	0.45	27.19	59.95	0.0081	0.4881
13	TACGTTCATGGATGTATG	0.51	31.09	60.90	0.0092	0.5582
14	TACAATCACGGATGTATG	0.52	32.21	61.59	0.0094	0.5783
15	TACAATCAAGGATGTATG	0.72	32.67	45.31	0.0129	0.5866
16	TACAATCCTGAATGTATG	0.71	37.52	52.54	0.0128	0.6735
17	TACATTCTCGGATGTATG	0.81	38.38	47.59	0.0145	0.6890
18	TACAACCGTGACTGTATG	1.53	39.48	25.82	0.0274	0.7087
19	TACATCCGTGGCTGTATG	1.27	42.79	33.70	0.0228	0.7683
20	TACATTCATGGATGTATC	2.24	46.14	20.55	0.0403	0.8283

TtgR

Name	Sequence	Repressed	Induced	FI	Repressed ratio to apFab71	Induced ratio to apFab71
Native	TATTTACAAACAACCATGAATGTAAGTA	0.06	3.15	52.63	0.0011	0.0566
1	TACGTACTTGACTGTATG	0.15	10.29	70.20	0.0026	0.1847
2	CTCCATACATCCGTGAATG	0.15	14.87	102.06	0.0026	0.2669
3	TACATACACGCGTGTTTG	0.19	18.06	97.50	0.0033	0.3242
4	GGACATACATTCGGGATTG	0.60	21.28	35.29	0.0108	0.3821
5	TACATACATGACTGTTTG	0.88	25.72	29.25	0.0158	0.4618
6	TACCTGCACGGATGTATG	0.34	26.70	78.80	0.0061	0.4793
7	CATACATTCACGATTG	0.34	26.87	78.42	0.0062	0.4824
8	TACATACGTTCATGGATG	0.33	27.00	82.22	0.0059	0.4848
9	AACCTTCATGGATGTATG	0.38	30.39	79.85	0.0068	0.5456
10	CTTACGTTCATGAATG	0.51	33.64	65.37	0.0092	0.6040
11	TACCTTCGTGTACGTATG	0.65	33.79	51.73	0.0117	0.6067
12	CATACATTCTTGCATG	0.50	34.50	68.67	0.0090	0.6194
13	TACATTCATGGAAGTATG	0.56	35.25	62.40	0.0101	0.6328
14	TACCTTCGTGTACGTATG	0.46	35.38	76.44	0.0083	0.6351
15	CATACATCCTTGGTTG	0.49	38.13	77.52	0.0088	0.6845
16	ACATACATCCTTGCATG	1.30	38.34	29.38	0.0234	0.6884
17	TACATTCCGGAATGTTTG	0.69	39.48	56.98	0.0124	0.7088
18	CATACATCCGTGCATG	0.53	40.39	76.04	0.0095	0.7252
19	TACCTTCTTGCATGTATG	0.75	45.35	60.42	0.0135	0.8143
20	AACATCCATGGAGGTATG	1.24	49.87	40.37	0.0222	0.8954

NalC

Name	Sequence	Repressed	Induced	FI	Repressed ratio to apFab71	Induced ratio to apFab71
Native	AGAACTGTATCGTACAGTACT	1.359932807	11.5109786	8.464	0.0018	0.0081
1	TGTGTACCGTACCGTACAG	0.111	6.260	56.364	0.0020	0.1124
2	ACTGTACTGTACAGTACGG	0.126	6.814	53.959	0.0023	0.1223
3	TAGTGTACTGGCCAGTACA	0.683	7.395	10.835	0.0123	0.1328
4	TGTGTACTGTCCAGTACAG	0.518	8.147	15.728	0.0093	0.1463
5	GATGTACTGTACAGTACAG	0.200	8.475	42.289	0.0036	0.1521
6	GAGTGTACCGGTCAGTACA	1.554	12.277	7.901	0.0279	0.2204
7	ACTGTACTGGGGAGTACAC	1.360	12.408	9.124	0.0244	0.2228
8	TGTGTACTAGGGAGTACAG	0.249	12.542	50.403	0.0045	0.2252
9	AGTGTACCGGTGAGTACAC	1.246	12.927	10.375	0.0224	0.2321
10	AAGTGTACCGGTCAGTACA	1.156	12.937	11.194	0.0207	0.2323
11	AGTGTACTGTATGGTACGG	2.252	13.796	6.125	0.0404	0.2477
12	GTGTACTGTACAGTACAG	0.497	14.452	29.070	0.0089	0.2595
13	AGTGTACTAGATAGTACAG	3.540	17.910	5.059	0.0636	0.3216
14	TAAGTGTACTGGCCAGTAC	2.698	19.780	7.332	0.0484	0.3551
15	AGTGTACTGATGAGTACA	2.203	20.101	9.123	0.0396	0.3609
16	GTACTGTACAGTACAG	0.523	20.853	39.891	0.0094	0.3744
17	GTGTACTGTACAGTACGG	0.433	21.719	50.143	0.0078	0.3899
18	GTGTACTGTATGGTACAG	0.635	26.918	42.365	0.0114	0.4833
19	GTGTACTGAACAGTACAG	2.228	27.767	12.464	0.0400	0.4985
20	GTACTGTACAGTACGG	1.413	29.774	21.072	0.0254	0.5345

Table S2: DNA sequences of aTFs and gel shift binding oligos used in the study

PmeR	ATGGTGCGTCGTACCAAGGAAGAGGCGCAGATCACCCGTAGCCAAATTCTGGAGGCGGCGG<br>AACAGGCGTTCTATGAGCGTGGTGTGGCGCGTACCACCCTGGCGGATATTGCGACCCTGGC<br>GGGTGTTACCCGTGGCGCGATTTATTGGCACTTTAACAACAAAGCGGACCTGGTTCAGGCGA<br>TGCTGGATAGCCTGCAAGAGCCGCTGGACGAAATGGCGCAGGCGAGCCAAAGCGAAGACG<br>AGGAAGATCCGCTGGGCTGCATGCGTAACCTGCTGATCCACCTGTTCCATGAGCTGGCGCTG<br>GACCCGAAGACCCGTCGTATCAACGAAATTCTGTTCCACAAATGCGAGTTTACCGACGAAA<br>TGTGCGATTTTCGTCGTCAGCGTCAAGATAACGCGATCCAGTGCCACGACCGTATTACCCTG<br>GGTCTGAGCAACGCGGTGCGTCAGGGTCAACTGCCGCAAGAACTGGATACCGGTCGTGCG<br>GCGGTGGCGCTGTTCAGCTACGTTAACGGCATCATTTATCAGTGGCTGCTGGTTCCGGACAG<br>CTTTAGCCTGCCGGCGGAGGCGGAACAACTGGTGGACGTTTGCCTGGATATGCTGCGTTTC<br>AGCCCGACCCTGCGTGTGAAGAACAGCCCGGAACTGAACGTTGAGCGTGAACGTCTG
TtgR	ATGGTGCGTCGCACCAAAGAAGAAGCACAGGAAACGCGTGCGCAGATTATCGAAGCGGCC<br>GAACGCGCGTTTTATAAACGTGGTGTGGCACGTACCACGCTGGCAGATATTGCAGAACTGG<br>CAGGTGTTACCCGCGGTGCAATCTACTGGCATTTCAACAATAAAGCCGAACTGGTTCAGGCA<br>CTGCTGGATTCTCTGCACGAAACGCATGATCACCTGGCCCGTGCAAGCGAATCTGAAGATG<br>AACTGGACCCGCTGGGCTGCATGCGCAAACTGCTGCTGCAGGTGTTTAACGAACTGGTTCT<br>GGATGCACGTACCCGTCGCATTAATGAAATCCTGCATCACAAATGCGAATTTACGGATGATAT<br>GTGTGAAATTCGTCAGCAGCGCCAGAGCGCCGTGCTGGATTGTCATAAAGGTATCACCCTG<br>GCACTGGCAAACGCAGTTCGTCGCGGTCAGCTGCCGGGTGAACTGGATGTGGAACGCGCA<br>GCGGTTGCGATGTTTGCCTATGTGGATGGCCTGATTGGTCGTTGGCTGCTGCTGCCGGATAG<br>TGTTGATCTGCTGGGCGATGTGGAAAAATGGGTTGATACCGGTCTGGATATGCTGCGTCTGA<br>GCCCGGCGCTGCGCAAA
NalC	ATGAACGATGCGAGCCCGCGTCTGACCGAGCGTGGCCGTCAGCGTCGTCGTGCGATGCTGG<br>ATGCGGCGACCCAAGCGTTCCTGGAGCACGGTTTTGAAGGCACCACCCTGGATATGGTGAT<br>CGAACGTGCGGGTGGTAGCCGTGGCACCCTGTACAGCAGCTTCGGTGGCAAAGAGGGTCT<br>GTTTGCGGCGGTTATTGCGCACATGATCGGTGAAATTTTTGACGATAGCGCGGACCAGCCGC<br>GTCCGGCGGCGACCCTGAGCGCGACCCTGGAGCACTTCGGTCGTCGTTTTCTGACCAGCCT<br>GCTGGACCCGCGTTGCCAGAGCCTGTATCGTCTGGTGGTTGCGGAAAGCCCGCGTTTCCCG<br>GCGATCGGCAAGAGCTTTTACGAGCAAGGCCCGCAGCAAAGCTATCTGCTGCTGAGCGAAC<br>GTCTGGCGGCGGTGGCGCCGCACATGGACGAGGAAACCCTGTACGCGGTTGCGTGCCAGTT<br>CCTGGAGATGCTGAAGGCGGACCTGTTTCTGAAAGCGCTGAGCGTGGCGGATTTCCAACCG<br>ACGATGGCGCTGCTGGAGACCCGTCTGAAACTGAGCGTTGATATCATTGCGTGCTATCTGGA<br>ACACCTGAGCCAGAGCCCGGCGCAAGGC
PmeR oligo	cy5-CGGGTGTTTACAAACAACCGCGAATGTAAGTATATTCCTTGACACTTCTGCCCAGGCGA<br>G
TtgR oligo	cy5-CTGTTCGTATTTACAAACAACCATGAATGTAAGTATATTCGACACTTCTGCCCAGGCGAG
NalC oligo	cy5-GGTCATTTAAGAACTGTATCGTACAGTACTGTTTTGGCAAGACACTTCTGCCCAGGCGA<br>G
16N Library	TGCGACGGTCTCACTGAGGCGCGCCTTGACANNNNNNNNNNNNNNNNTATAATAGATTCAT<br>GATGAGAATTCATTAAAGAGGAGAAAGGT
17N Library	TGCGACGGTCTCACTGAGGCGCGCCTTGACANNNNNNNNNNNNNNNNNTATAATAGATTCA<br>TGATGAGAATTCATTAAAGAGGAGAAAGGT
18N Library	TGCGACGGTCTCACTGAGGCGCGCCTTGACANNNNNNNNNNNNNNNNNNTATAATAGATT<br>CATGATGAGAATTCATTAAAGAGGAGAAAGGT
19N Library	TGCGACGGTCTCACTGAGGCGCGCCTTGACANNNNNNNNNNNNNNNNNNNTATAATAGAT<br>TCATGATGAGAATTCATTAAAGAGGAGAAAGGT

Table S3: Plasmid maps of plasmids used in this study

Sensor plasmid carrying aTF	TTGACAATTAATCATCCGGCTCGTATAATAGATTCATTAGAGTCTAG<br>AGAAAGACAGGATTAAC[insert aTF sequence here]<br>ATTAGCAGAAAGTCAAAAGCCTCCGACCGGAGGCTTTTGACTAAA<br>ACTTCCCTTGGGGTTATCATTGGG<br><br>apFab6 - BBa_J61132 - aTF - tonB termnator; all sensor plasmids carry spec^{R}; the origin of replication for plasmids carrying PmeR and TtgR is a mutant SC101 (high copy); the origin of replicatin for the plasmid carrying NalC is p15a (medium ot low)
pXL-3	TTGACATCGCATCTTTTTGTACCTATAATAGATTCATGATGAGAATT<br>CATTAAAGAGGAGAAAGGTATGCGTAAAGGCGAAGAGCTGTTCA<br>CTGGTTTCGTCACTATTCTGGTGGAACTGGATGGTGATGTCAACG<br>GTCATAAGTTTTCCGTGCGTGGCGAGGGTGAAGGTGACGCAACT<br>AATGGTAAACTGACGCTGAAGTTCATCTGTACTACTGGTAAACTG<br>CCGGTACCTTGGCCGACTCTGGTAACGACGCTGACTTATGGTGTT<br>CAGTGCTTTGCTCGTTATCCGGACCACATGAAGCAGCATGACTTC<br>TTCAAGTCCGCCATGCCGGAAGGCTATGTGCAGGAACGCACGAT<br>TTCCTTTAAGGATGACGGCACGTACAAAACGCGTGCGGAAGTGA<br>AATTTGAAGGCGATACCCTGGTAAACCGCATTGAGCTGAAAGGC<br>ATTGACTTTAAAGAAGACGGCAATATCCTGGGCCATAAGCTGGAA<br>TACAATTTTAACAGCCACAATGTTTACATCACCGCCGATAAACAA<br>AAAAATGGCATTAAAGCGAATTTTAAAATTCGCCACAACGTGGA<br>GGATGGCAGCGTGCAGCTGGCTGATCACTACCAGCAAAACACTC<br>CAATCGGTGATGGTCCTGTTCTGCTGCCAGACAATCACTATCTGA<br>GCACGCAAAGCGTTCTGTCTAAAGATCCGAACGAGAAACGCGAT<br>CACATGGTTCTGCTGGAGTTCGTAACCGCAGCGGGCATCACGCAT<br>GGTATGGATGAACTGTACAAATAATCGCCAGCAGGCCTTTTTATTT<br>GGGGGAGAGGGAAGTCATGAAAAAACTAACCTTTGAAATTCGAT<br>CTCCACCACATCAGCTCTGAAGCAACGTAAAAAAACCCGCCCCG<br>GCGGGTTTTTTTATACCCGTAGTATCCCCACTTATCTACAATAGCT<br>GTCCTTAATTAATCTAGA<br><br>apFab71 - Bujard RBS - sfGFP - terminator; the plasmid carry kan^{R}; the origin of replication for the plasmid is ColE1 (high copy)

Table S4: Number of unique sequences of 16, 17, 18 and 19 base pair (bp) spacers in PmeR, TtgR and NalC promoter libraries after *in vitro* enrichment

TF	16bp	17bp	18bp	19bp
PmeR	274685	383185	408077	435910
TtgR	347150	476816	559574	563217
NalC	123320	180071	204383	261711

Table S5: Fluorescence activated cell sorting binning conditions for PmeR, TtgR and NalC promoter libraries. Bins were numbered from high GFP fluorescence to low GFP fluorescence with increasing bin number for each library. Low and high boundaries of bins and population medium were recorded in GFP fluorescence. Each bin's population size is reflected by its percentage of the whole library

Promoter Library	Bin Number	Fluorescence Low Boundary	Fluorescence High Boundary	Medium Fluorescence	Percentage of Library
PmeR	1	124974	781519	153543	6.18
PmeR	2	34176	120534	58372	54.96
PmeR	3	9349	32967	23909	21.83
PmeR	4	968	9016	3700	14.31
TtgR	1	65351	409922	88776	5.69
TtgR	2	26562	63029	37671	20.12
TtgR	3	12469	25621	18159	22.53
TtgR	4	5253	12025	8091	23.63
TtgR	5	1601	5067	3159	19.09
TtgR	6	151	1601	1111	5.12
NalC	1	87183	509866	121512	8.41
NalC	2	26561	84081	41109	28.32
NalC	3	9348	25620	16349	32.79
NalC	4	3410	9016	6028	18.5
NalC	5	869	3289	2226	7.84
NalC	6	167	3172	2047	8.31

Table S6: **Comparison of length of gap-filled sequences generated by different multiple sequence alignment tools using default parameter settings.** The set of 36 functional operator sequences and 157 non-functional operator sequences (in fasta format) corresponding to 'TtgR' was used as the input.

Method/ Tool	Length of gap-filled sequence	References
T-coffee	27	(Notredame, Higgins, & Heringa, 2000)
MAFFT	31	(Katoh, 2002)
Clustal Omega	32	(Sievers et al., 2014)
MUSCLE	40	(Edgar, 2004)

Table S7: **Performance metrics for models built using support vector regression with radial basis function for the three different transcription factors datasets**

Transcription factor	No. of data-points		No. of features		Time taken[d] (in min)	Best parameter[e]		R^2	RMSE	Spearman Rank Coefficient
	Inducible[a]	Non-inducible[b]	Initial	Final[c]		C	gamma			
TtgR	36	41	92	20	51.6	10	1	0.88	3.564	0.879[f]
PmeR	25	22	84	23	33.6	100	0.1	0.84	7.138	0.889[f]
NalC[g]	62	61	124	80	164.3	10	0.1	0.83	2.572	0.928[f]

[a] - sequences hits obtained from FACS assay

[b] - top one-hundredth percent of most abundant sequences from *in vitro* binding assay and a null sequence (or a string of gap-characters). Fold induction ratio was assumed to be 1 for this set.

[c] - no. of features chosen to build final ensemble model based on 100 runs of simulated annealing across bootstrap sampled datasets

[d] - computational run time when run in parallel across 23 CPUs (Intel Xeon 2.4 GHz processors) with cache size capped at 20 GB of RAM space

[e] - found during grid search based hyper-parameter tuning over the range ~ 0.001 to 2048 and ~ 0.00003 to 8 for C and gamma, respectively

[f] - *p*-val $<< 10^{-6}$

[g] - As NalC dataset had high degree of correlated features, a less stringent threshold was used for feature selection

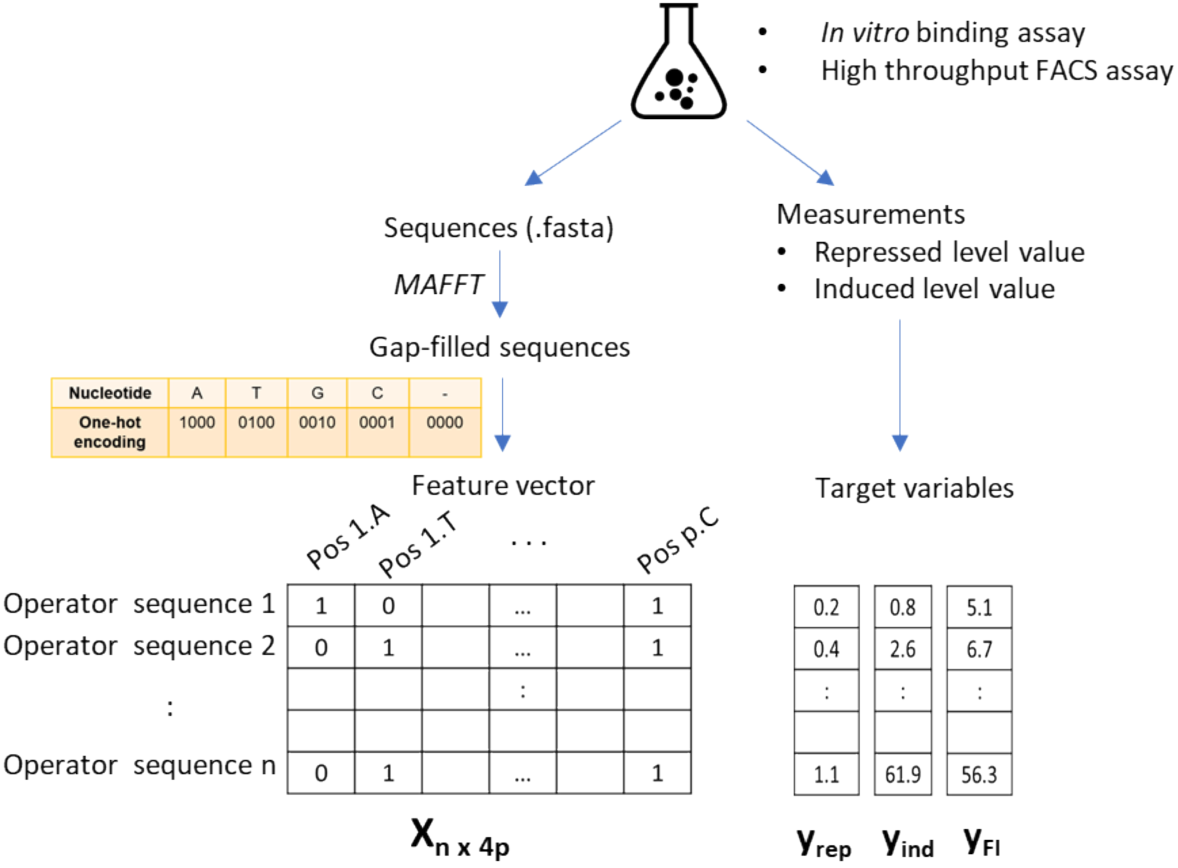

Figure S9: Data pre-processing and generation of design matrix, X and target variables (yrep, yind, & yFI).
